# Artificial Intelligence Platform Architecture for Hospital Systems: Systematic Review

**DOI:** 10.2196/79788

**Published:** 2025-12-17

**Authors:** Musitapa Maimaitiaili, Yiershatijiang Jiamaliding, Guangle Dai, Hui Xiao, Warisijiang Kuerbanjiang, Yuexiong Yi

**Affiliations:** 1 Department of Gynecology, Zhongnan Hospital of Wuhan University, #169, Donghu Road, Wuchang District, Wuhan, Hubei, 430071, China, 86 15671669885, 86 02767813142; 2 The Second Clinical Hospital, Wuhan University, Wuhan, China; 3 Information Center, Zhongnan Hospital of Wuhan University, Wuhan, China

**Keywords:** artificial intelligence, hospital AI platform, 5-layer architecture, health care digitization, AI implementation framework

## Abstract

**Background:**

The construction of artificial intelligence (AI) platforms in hospitals is the backbone of the revolution in health care. While traditional hospital information systems have facilitated digitalization, they are still limited by data silos, fragmented workflows, and insufficient clinical intelligence that impede organizations from realizing the promise of data-led decision-making.

**Objective:**

This study aimed to derive a hospital-specific 5-layer architecture (infrastructure, data, algorithm, application, and security and compliance) and to systematically review the evidence mapped onto the 5-layer framework to assess its applicability.

**Methods:**

A systematic literature search was performed in Web of Science, Embase, PubMed, and Scopus from inception to May 2025. The review followed the PRISMA (Preferred Reporting Items for Systematic Reviews and Meta-Analyses) guidelines. Studies were screened and selected for full-text review by two independent reviewers. We included peer-reviewed empirical studies describing hospital-based AI implementations across clinical domains. Reviews, commentaries, and purely technical bench studies without hospital context and non-English literature were excluded. Quality assessment of the identified papers was conducted using the Critical Appraisal Skills Programme tool. Using a 0 to 5 point ordinal maturity scale of 5 layers, we conducted a structured mapping with quantitative mapping, weighted co-occurrence analysis, weighted Jaccard similarity, and thematic synthesis with examples.

**Results:**

In total, 29 studies met the eligibility criteria and included work specifically in emergency, radiology, routine imaging, chronic disease, and multihospital platform work, conducted in 11 countries. On average, the application (mean 3.17, SD 0.85) and data (mean 3.00, SD 0.76) layers demonstrated the highest maturity, followed by algorithm (mean 2.79, SD 0.77) and infrastructure (mean 2.79, SD 1.70). The security and compliance layer showed the lowest and most variable maturity (mean 1.69, SD 1.89). Weighted co-occurrence and Jaccard analyses revealed strong interconnections among data, algorithm, and application (Jaccard=0.80‐0.89), forming a technical core, whereas security and compliance exhibited weak alignment (0.43‐0.46).

**Conclusions:**

Our review excluded non-English and gray literature, which may limit comprehensiveness. The ordinal maturity scoring may still simplify the contextual complexity of hospital AI implementations. Our synthesis validates a 5-layer hospital AI platform architecture, grounded in both theoretical frameworks and empirical evidence. The findings highlight that while clinical feasibility is achievable, sustainable hospital-wide AI requires stronger investment in infrastructure, data governance, and compliance.

## Introduction

### Background

Artificial intelligence (AI) is often seen as a game changer for health care, yet many hospitals face challenges in scaling it beyond pilot projects due to limited resources and infrastructure. Rising chronic disease burden due to aging populations and increased demand for personalization has exacerbated these challenges [1]. Traditional hospital information systems (Hospital Information System [HIS], Laboratory Information Systems [LIS], and Picture Archiving and Communication System [PACS]) have become administrative tools without analytic or decision-support value [2]. Wide varieties of multimodal health data are now available along with advanced AI methods, such as image recognition, natural language processing, and predictive analytics, that are maturing rapidly [3]. However, most hospitals face difficulties in integrating these approaches within routine clinical workflows [4].

### Research Gap

Many hospitals have not built a system-level roadmap for scaling up and governance structure to support the use of AI pilot projects [5]. Fragmented infrastructures worsen this challenge: siloed PACS and LIS [6] limited interoperability across electronic health record (EHR) vendors due to inconsistent Health Level 7 (HL7) Fast Healthcare Interoperability Resources (FHIR) adoption [7], and heterogeneous Internet of Things (IoT) data streams [8]. Reported implementations typically target isolated tasks, such as radiology computer-aided diagnosis [9] or triage algorithms. Focusing on isolated tasks does not facilitate enterprise-wide implementation of AI, as it fails to address the need for integrated and scalable solutions. In this context, hospital executives do not have sensible roadmaps to translate fragmented digital environments into integrated AI platforms that are based on evidence [10].

### Existing Frameworks and Development of a Hospital-Specific Architecture

The hospital system-wide AI platform should be built over an existing framework and not a new one. To build a strong theory base, 4 representative frameworks were reviewed: (1) the Healthcare Information and Management Systems Society Digital Health Framework [11], (2) the World Health Organization (WHO) HIS framework [12], (3) sociotechnical theory [13], and (4) the National Institute of Standards and Technology (NIST) Big Data Reference Architecture (BDRA) [14]. Each offers distinct perspectives. The Healthcare Information and Management Systems Society emphasizes digital maturity and interoperability; the World Health Organization HIS highlights governance and service delivery; sociotechnical theory stresses the interaction of people, processes, and technologies; and BDRA provides a detailed blueprint for big data infrastructures. Comparison shows overlap in infrastructure and data integration, but divergences in analytics, governance, and algorithm lifecycle (Table 1).

**Table 1.** Original layer structures of 4 foundational frameworks.

Layer	HIMSS^a^ digital health framework	WHO^b^ HIS^c^ framework	Sociotechnical theory	NIST^d^ BDRA^e^
Layer 1	Infrastructure and interoperability	ICT^f^ resources	Technical subsystem	Infrastructure and platform services
Layer 2	Data exchange and integration	Health data sources and data flow	Information subsystem	Data pipeline
Layer 3	Analytics and predictive insights	Health services delivery	Organizational processes and workflows	Analytics and big data applications
Layer 4	Person-enabled health	Policy, ethics, and regulation	People–organization interactions and accountability	Application services and outputs
Layer 5	Governance and workforce	—^g^	—	Governance, security, compliance services

a HIMSS: Healthcare Information and Management Systems Society.

b WHO: World Health Organization.

c HIS: hospital information system.

d NIST: National Institute of Standards and Technology.

e BDRA: Big Data Reference Architecture.

f ICT: information and communication technology.

g Not available.

**Figure 1.**
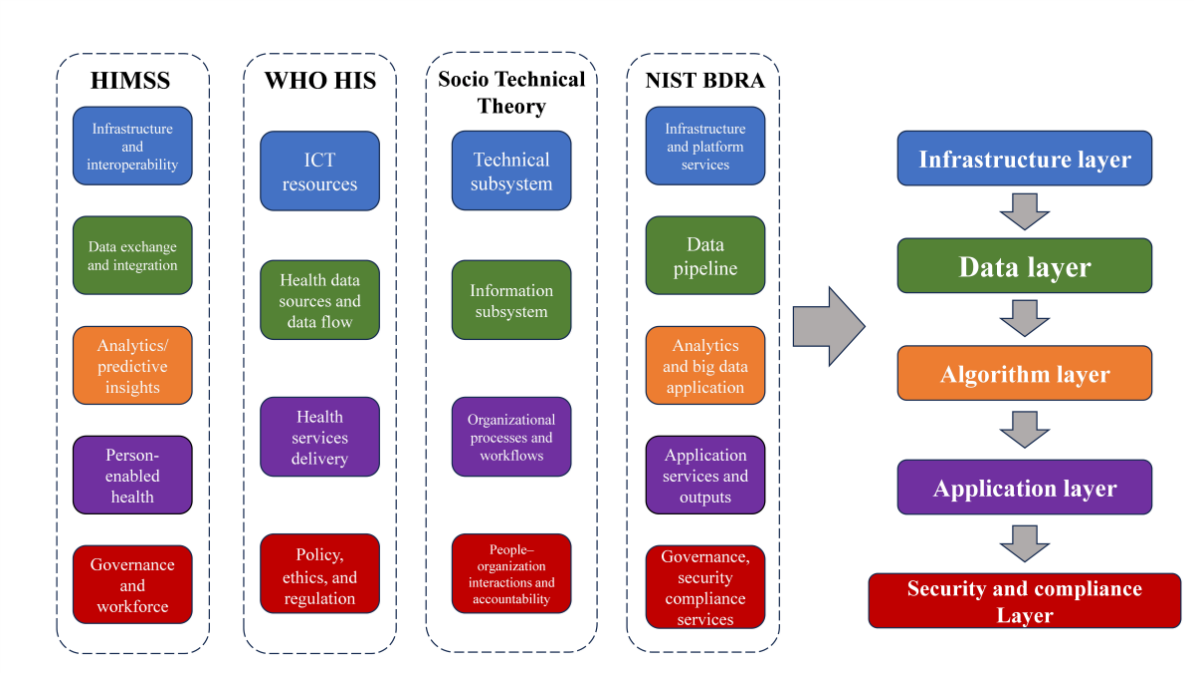
Derivation of the 5-layer architecture from 4 foundational frameworks. Constructs from the HIMSS digital health framework, the WHO HIS framework, sociotechnical theory, and the NIST BDRA were compared, merged, and normalized to generate a unified 5-layer model (infrastructure, data, algorithm, application, and security and compliance). Color coding highlights conceptually equivalent constructs across frameworks: blue represents infrastructure; green represents data; orange represents algorithm and analytics; purple represents applications; and red represents governance, security, and compliance. BDRA: Big Data Reference Architecture; HIMSS: Healthcare Information and Management Systems Society; HIS: Health Information System; NIST: National Institute of Standards and Technology; WHO: World Health Organization.

Despite important similarities, none of these frameworks fully address the unique requirements for hospital-wide AI implementation, particularly in terms of comprehensive integration and lifecycle management. To bridge this gap, we applied a merge-and-normalize approach by extracting core constructs, clustering overlapping elements, and adding components related to AI where the original was missing, especially on algorithm lifecycle management. This process produced 5 interoperable layers tailored to hospital AI contexts: infrastructure, data, algorithm, application, and security and compliance (Figure 1). Specifically, (1) the infrastructure layer covers compute, storage, and network foundations; (2) the data layer ensures standards such as HL7 FHIR and Digital Imaging and Communications in Medicine, multimodal integration across EHR, LIS, and IoT, and quality management; (3) the algorithm layer introduces the AI lifecycle, including development, validation, monitoring, and operations; (4) the application layer emphasizes workflow integration, such as decision support, triage, and patient-facing tools; and (5) the security and compliance layer ensures privacy, accountability, and governance.

### Objectives

On the basis of identified gaps and synthesized frameworks, this study intends to propose a 5-layer AI platform architecture specific to hospitals, to systematically review the empirical studies by mapping evidence onto the framework, and to synthesize theoretical and empirical insights to offer practical advice for hospital administrators, policymakers, and developers who want to build a scalable, secure, and clinically integrated AI ecosystem. Two research questions (RQs) guide this process: (1) RQ1: What empirical evidence supports each layer of the proposed 5-layer architecture? and (2) RQ2: How do the interrelationships among layers reveal strengths and gaps in hospital-level AI research?

## Methods

### Study Design

This systematic review followed the PRISMA (Preferred Reporting Items for Systematic Reviews and Meta-Analyses) 2020 and PRISMA-P (Preferred Reporting Items for Systematic Review and Meta-Analysis Protocols) guidelines [15] and was prospectively registered in PROSPERO (CRD420251133590). A completed PRISMA checklist is provided in Checklist 1. The 5-layer architecture derived from existing frameworks was adopted as an a priori coding structure for evidence mapping for identifying studies on hospital AI implementation and aligning their findings with this architecture to link theoretical constructs with real-world practice.

### Search Strategy

A comprehensive search was conducted in Web of Science, Embase, PubMed, and Scopus from their inception to May 23, 2025. To maximize sensitivity, the strategy combined free-text keywords with controlled vocabulary terms. Search terms included “Hospital Management,” “Healthcare Management,” “Hospital Operations,” “Healthcare Administration,” “Medical Administration,” “Hospital Information Systems,” “AI Deployment,” “AI Implementation,” “AI Integration,” “Artificial Intelligence,” “AI Applications,” “Large Language Models,” “Transformer Applications,” “Hospital Operations Optimization,” “Clinical Workflow Improvement,” “Resource Allocation,” and “Patient Flow Management.” The full search strings for all databases are detailed in Multimedia Appendix 1.

In addition, reference lists of all included articles were manually screened to identify further eligible studies not captured by the electronic search.

### Eligibility Criteria

Included studies were in a hospital setting and documented real-world AI use (eg, machine learning, deep learning, natural language processing, predictive analytics, and expert systems) that presented measurable outcomes, including enhanced efficiency, decreased costs/errors, optimized resource usage, improved patient experience, or accurate diagnosis/decision-making enhancement. Studies that did not use AI were excluded, along with reviews, commentaries, conference abstracts, non-English studies, and studies without an abstract. The summary of inclusion and exclusion criteria can be seen in Textbox 1.

Textbox 1. **Inclusion and exclusion criteria**

Inclusion criteria

- Studies conducted in hospital environments (operation rooms, decision-making, emergency services, patient flow management, resource allocation)
- Studies involving artificial intelligence technologies such as machine learning, deep learning, natural language processing, predictive analytics, or expert systems
- Studies describing real-world, pilot, or simulation-based evaluations conducted in a hospital context using real or representative institutional data
- Studies reporting outcomes related to efficiency improvement, cost reduction, error reduction, resource utilization, patient satisfaction, or decision-making accuracy
- Applications targeting hospital operations, planning, scheduling, cost control, risk prediction, patient care, or workflow optimization

Exclusion criteria

- Studies set entirely outside of hospitals
- Studies that mention artificial intelligence (AI) only conceptually or theoretically, without implementation details
- Studies focusing only on general information systems without AI integration
- Studies without measurable or reported outcomes
- Non-English study
- Review or commentaries or meeting abstract or letters
- No abstract exists

### Screening

Two reviewers independently screened titles and abstracts, removed duplicates, and assessed preliminary eligibility. The full-text evaluation of the relevant studies was conducted based on the inclusion and exclusion criteria. Reviewers were not blinded to study authorship or outcomes. Discrepancies were resolved by discussion and consensus. The reliability of screening was evaluated through the Cohen κ statistic, and the results were summarized in a 2×2 contingency table.

### Data Extraction and Synthesis

Data were extracted using a standardized form, including bibliographic details, study design, clinical domain, objectives, clinical applications, and limitations. Explicit coding definitions were used to enable mapping of each study to the 5-layer architecture (infrastructure, data, algorithm, application, and security and compliance). To promote transparency, the coding criteria for each layer are given in Table 2. Each layer was scored on a 0 to 5 point ordinal maturity scale conceptually aligned with the Capability Maturity Model Integration framework [16] with level definitions and examples detailed in Table 3. Two reviewers independently performed the scoring, and discrepancies were resolved by consensus.

**Table 2.** Coding definitions for the 5-layer architecture.

Layer	Coding definition
Infrastructure	Assign when the evidence describes compute, storage, network, cloud/edge topology, containerization/orchestration, or system-level integration capacity that hosts or executes AI^a^ workloads (eg, data centers, GPU pools, hybrid cloud, and enterprise integration with HIS^b^/PACS^c^/LIS^d^).
Data	Assign when the evidence concerns data sources and flows, standards/interoperability (HL7^e^ FHIR^f^, DICOM^g^, and terminologies), identity/linkage (EMPI^h^), multimodal integration (EHR^i^, imaging, monitors/IoT^j^, notes, and omics), data quality, lineage/provenance, or deidentification that make data AI ready.
Algorithm	Assign when the evidence covers AI/ML^k^ methods and lifecycle: model development/training, internal or external validation, performance metrics, monitoring/drift, retraining, explainability, or federated or edge learning, irrespective of where the model will later be used.
Application	Assign when the evidence demonstrates embedding AI into clinical or operational workflows, including CDS^l^, triage/priority, worklist optimization, patient-flow/bed management, scheduling, or patient-facing tools; focus is on use in work (UI^m^/UX^n^, pathway location, task changes).
Security and compliance	Assign when the evidence addresses privacy/security controls (access control, encryption, OAuth/HTTPS, audit logs, blockchain audit), consent and data use governance, regulatory/ethical compliance (HIPAA^o^, GDPR^p^, local policies), or model governance/accountability.

a AI: artificial intelligence.

b HIS: hospital information system.

c PACS: picture archiving and communication system.

d LIS: laboratory information systems.

e HL7: Health Level 7.

f FHIR: Fast Healthcare Interoperability Resources.

g DICOM: Digital Imaging and Communications in Medicine.

h EMPI: Enterprise Master Patient Index.

i EHR: electronic health record.

j IoT: Internet of Things.

k ML: machine learning.

l CDS: clinical decision support.

m UI: user interface.

n UX: user experience.

o HIPAA: Health Insurance Portability and Accountability Act.

p GDPR: General Data Protection Regulation.

**Table 3.** The 0 to 5 point maturity scale and the Capability Maturity Model Integration (CMMI) framework.^a^

Score	CMMI level	CMMI descriptor	Hospital AI^b^ platform context
0	—^c^	—	No evidence of this layer in the study; the layer is not addressed or discussed.
1	Level 1: initial	Processes are ad hoc, chaotic, and unstructured. Success depends on individual effort.	Conceptual or pilot-level mention; ad hoc implementation without governance or integration.
2	Level 2: managed	Basic project management processes established to track cost, schedule, and functionality.	Layer partially implemented or managed within a limited scope (eg, single department).
3	Level 3: defined	Processes are documented, standardized, and integrated into organizational practice.	Layer implemented with defined workflows, policies, or institutional governance structures.
4	Level 4: quantitatively managed	Organization uses quantitative data to control and monitor processes.	Layer performance is monitored with metrics; multiple departments coordinate and share data.
5	Level 5: optimizing	Focus on continuous improvement and innovation based on quantitative feedback.	Fully institutionalized, hospital-wide, or cross-site implementation with feedback loops and continuous optimization.

a The 0-5 maturity scoring system was conceptually aligned with the CMMI framework (Software Engineering Institute, Carnegie Mellon University), with “0” added to capture the absence of evidence in individual studies.

b AI: artificial intelligence.

c Not applicable.

Quantitative synthesis summarized the average maturity scores and SDs for each layer across studies and by study design. Weighted co-occurrence matrices were computed to quantify the cumulative maturity shared between layers, whereas weighted Jaccard similarity indices measured the strength of cross-layer coupling. These metrics were visualized in heatmaps to illustrate maturity distribution and interlayer relationships. Qualitative synthesis identified thematic patterns, gaps, and illustrative cases to demonstrate interactions among layers in real-world contexts.

### Quality Assessment

The Critical Appraisal Skills Programme (CASP) tool [17] was applied to assess the quality of included studies because it can be used across various study types. Using checklists for qualitative, quantitative, and mixed methods research, the Critical Appraisal Tools (CATS) tool is used more often than other important assessments, such as the Joanna Briggs Institute, Cochrane, and GRADE. All articles were evaluated for methodology, reliability, interpretation, and usability.

### Statistical Analysis

Descriptive statistics were used to summarize the maturity distribution of the 5 layers, expressed as mean scores and SDs across all included studies. Studies were further stratified by methodological category to compare average maturity levels within and across study designs.

Cross-layer relationships were analyzed using weighted co-occurrence and weighted Jaccard similarity metrics derived from the 0 to 5 maturity scores.

The weighted co-occurrence is defined as follows:


$$W_{co}\left(A,B\right)=\sum_{i=1}^{n}\text{min}\left(A_{i},B_{i}\right)$$

where $A_{i}$and $B_{i}$represent the maturity scores (0‐5) assigned to layers A and B in study i.

The weighted Jaccard similarity is defined as follows:


$$J_{w}\left(A,B\right)=\frac{\sum_{i=1}^{n}\text{min}\left(A_{i},B_{i}\right)}{\sum_{i=1}^{n}\text{max}\left(A_{i},B_{i}\right)}$$

where $A_{i}$and $B_{i}$represent the maturity scores (0‐5) assigned to layers A and B in study i.

Interrater reliability during study selection was quantified using Cohen κ statistic, defined as follows:


$$\kappa =\frac{p_{o}-p_{e}}{1-p_{e}}$$

where $p_{o}$ is the observed agreement, and $p_{e}$ is the expected agreement by chance.

### Ethical Considerations

This systematic review does not involve human participants, identifiable patient data, or protected health information. All data analyzed in this review were obtained from publicly accessible publications. Therefore, an ethical review was not required under Zhongnan Hospital of Wuhan University’s secondary research policies. The study complied with the Declaration of Helsinki and institutional guidelines for secondary data use.

## Results

### Study Selection

**Figure 2.**
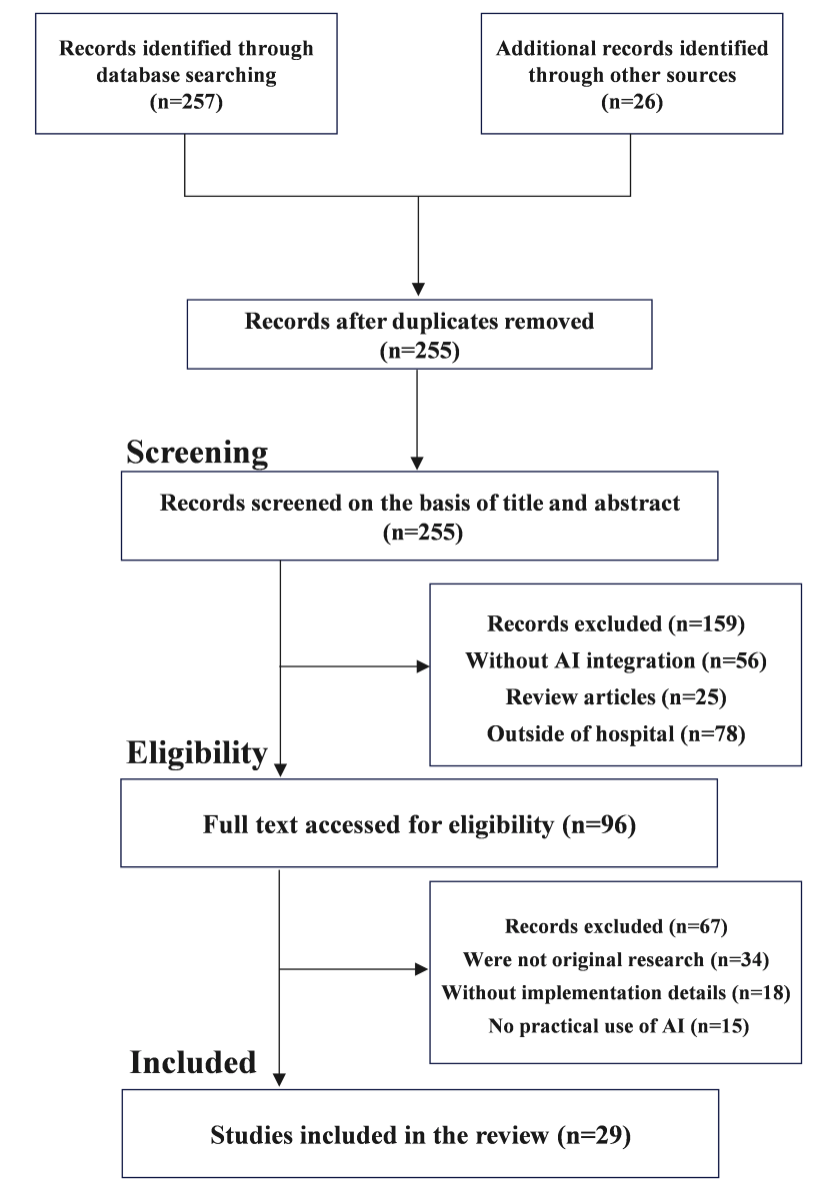
The search strategy for study inclusion is based on PRISMA (Preferred Reporting Items for Systematic Reviews and Meta-Analyses) guidelines. AI: artificial intelligence.

A total of 283 records were identified, of which 257 were from databases and 26 from other sources. After removing duplicates, 255 records were available to undergo title and abstract screening. At this stage, 159 records were excluded, comprising 56 non-AI cases, 25 review articles, and 78 cases not completed in the hospital. Of 96 full texts assessed for eligibility, 67 were ruled out for the following reasons: not original research (n=34), lacked implementation details (n=18), or practical AI use was not described (n=15). In the end, a total of 29 studies [18-46] were included in the review, representing 10.2% of the 283 records. Figure 2 illustrates the selection process. Screening was done by 2 reviewers independently, who demonstrated high interrater reliability (Cohen κ=0.98), suggesting almost perfect agreement. The 2×2 contingency table is shown in Multimedia Appendix 2.

### Study Characteristics

The characteristics of the 29 included studies are summarized in Tables4  5. Clinical coverage was broad: nonspecific or cross-specialty applications were most frequent (10/29, 34.5%) [18-21,25-27,30,40,45], followed by radiology (6/29, 20.7%) [22,24,29,43,44,46], emergency medicine (5/29, 17.2%) [28,31,33,37,39], cardiology (2/29, 6.9%) [34,41], and gynecology (2/29, 6.9%) [23,38]. Surgery [36], chronic disease [35], nursing [32], and psychiatry [42] each contributed 1 study.

**Table 4.** Characteristics of included studies.

Author (year)		Country	Clinical domain	Study design	Objective	Clinical application	Limitations
Ahsen et al (2025) [29]		United States	Radiology	Economic evaluations	Provide guidance on integrating AI^a^ into mammography workflows by balancing tasks between radiologists and algorithms	Breast cancer detection and risk assessment	Costs associated with algorithms, false assessments, and litigation expenses from false negatives
Boussen et al (2024) [33]		France	Intensive care medicine	Clinical prediction	To evaluate the performance of SAPS 2 PLUS model compared to the original SAPS 2 model by incorporating heart rate complexity and diastolic blood pressure measurements	Predicting ICU^b^ patient survival	Potential biases in datasets and limited generalizability due to single-center data
Alam et al (2025) [18]		United States	Nonspecific	Diagnostic test study	To assess the accuracy and reliability of ChatGPT 4.0 in interpreting 24 h ABPM^c^ data in clinical settings	ChatGPT 4.0 for interpreting 24 h ABPM data	Limited research validating AI models against expert interpretations in real-world clinical scenarios
Areias et al (2024) [20]		United States	Nonspecific	Cohort study	To explore the impact of scaling care through AI on patient outcomes, engagement, satisfaction, and adverse events	AI tool integrated into the physical therapist clinical portal to streamline workflow and support decision-making	Limited research on the impact of AI scalability approaches in clinical outcomes for MSK^d^ rehabilitation
Chen and Miao (2025) [25]		China	Nonspecific	Cross-sectional study	To evaluate the impact and effectiveness of DeepSeek, an AI-driven diagnostic tool, deployed across 90 tertiary hospitals in China	Improving diagnostic accuracy, enhancing clinical decision support, automating medical image analysis, streamlining workflow processes	High initial investment costs, requirement for robust data infrastructure, potential resistance from health care professionals, variability in model performance across different settings
Farghaly and Deshpande (2024) [44]		United States	Radiology	Diagnostic test study	Develop a novel classification model to distinguish COVID-19 from viral pneumonia using chest x-ray images	Automated classification of chest x-ray images into normal, COVID-19, and viral pneumonia categories to assist in early detection and diagnosis	Dataset bias, model generalizability, interpretability. The dataset used may not fully capture the diversity of real-world clinical settings. Imaging protocol variations could affect model performance
Fairbairn et al [34] (2025)		United Kingdom	Cardiovascular science	Cohort study	To evaluate the impact of a national AI technology program on cardiovascular outcomes and its broader effects on the health system	Predicting cardiovascular risk, optimizing treatment strategies, improving patient management and follow-up, enhancing clinical decision support systems	Data privacy concerns, initial implementation costs, variability in data quality across different regions, potential resistance from health care professionals
Jaganathan and Natesan (2025) [23]		India	Gynecology	Diagnostic study	To develop and evaluate an integrated system using blockchain technology and explainable AI for the detection and management of polycystic ovary syndrome	Early detection of PCOS^e^, personalized treatment recommendations, secure data sharing, enhanced patient privacy	Initial setup costs, need for robust data infrastructure, potential resistance from health care professionals, complexity in integrating blockchain with existing systems
Muntasir et al (2023) [40]		United States	Nonspecific	Qualitative study	To evaluate the impact of AI-assisted technologies on optimizing laboratory workflows in hospitals and improving overall efficiency	Automating sample processing, optimizing test ordering and prioritization, predicting equipment maintenance needs, enhancing data management and reporting	Automating sample processing, optimizing test ordering and prioritization, predicting equipment maintenance needs, enhancing data management and reporting
Ju et al [32] (2025)		Korea	Nursing	Cohort study	To develop and evaluate a generative AI system that provides nursing diagnosis and documentation recommendations using virtual patient electrocardiogram data	Assisting in nursing diagnoses, automating documentation processes, improving clinical decision support, enhancing patient care quality	Initial setup costs, need for high-quality training data, potential resistance from nursing staff, variability in model performance across different settings
Klumpp et al (2021) [21]		Germany	Nonspecific	Qualitative study	To explore various application cases of AI in hospital health care settings and address the challenges faced during implementation in European hospitals	Predictive analytics for patient outcomes, clinical decision support systems, automated diagnostic tools, workflow optimization	Data privacy regulations (eg, GDPR^f^), initial investment costs, need for high-quality data, resistance from health care professionals, variability in model performance across different institutions
Le et al (2024) [39]		United States	Emergency medicine	Cohort study	To evaluate the impact of a ML^h^-enabled automated system for detecting LVO^g^ on transfer times and patient outcomes in primary stroke centers	Early detection of LVO, optimizing patient transfer protocols, improving clinical decision-making, reducing time to treatment	Initial setup costs, need for high-quality training data, potential resistance from health care professionals, variability in model performance across different populations
Li et al (2024) [45]		United States	Nonspecific	Qualitative study	To develop and evaluate TrajVis, a visual clinical decision support system that translates AI trajectory models into actionable insights for health care	Predictive analytics for patient trajectories, personalized treatment recommendations, workflow optimization, enhancing communication between clinicians	Initial setup costs, need for high-quality training data, potential resistance from health care professionals, complexity in interpreting AI-generated insights
Lin et al (2025) [43]		United States	Radiology	Cohort study	To evaluate the effectiveness of risk-stratified screening schedules using AI models in optimizing daily mammography recalls and improving patient outcomes	Risk stratification for personalized screening, optimizing recall scheduling, reducing unnecessary follow-ups, enhancing early detection of breast cancer	Initial setup costs, need for high-quality training data, potential resistance from health care professionals, variability in model performance across different populations
Novak et al (2021) [26]		United States	Nonspecific	Qualitative study	To explore how design thinking methodologies can be applied to health informatics projects, using insights from Project Health Design as a case study	Enhancing patient-centered care, improving user experience, fostering innovation in health IT solutions, promoting interdisciplinary collaboration	Limited generalizability due to case-specific nature, potential resistance from traditional health care structures, need for ongoing stakeholder engagement, challenges in integrating with existing systems
Nsubuga et al (2025) [31]		Uganda	Emergency medicine	Diagnostic test study	To evaluate the performance of ML models for trauma triage in low-resource settings and compare it with traditional triage methods	Automated trauma triage, predictive analytics for patient outcomes, optimizing resource allocation, improving clinical decision-making	Initial setup costs, need for high-quality training data, potential resistance from health care professionals, variability in model performance across different populations, challenges in low-resource settings
Pariso et al (2025) [19]		Italy	Nonspecific	Cross-sectional study	To evaluate the impact of integrating AI into energy management systems in Italian hospitals, focusing on efficiency improvements and cost savings	Energy consumption optimization, predictive maintenance, demand response, and reducing carbon footprint	Initial setup costs, need for high-quality data, potential resistance from facility managers, variability in model performance across different facilities, and integration with existing systems
Vignapiano et al (2025) [42]		Italy	Psychiatry	Cross-sectional study	To evaluate proximity-based solutions that integrate clinical and technological advances to optimize treatment for autism spectrum disorder	Personalized treatment plans, behavior monitoring, predictive analytics for symptom progression, and enhancing communication and social skills	Data privacy concerns, initial setup costs, need for high-quality training data, variability in model performance across different individuals, resistance from health care professionals and caregivers
Roppelt et al (2025) [30]		Germany	Nonspecific	Qualitative study	To explore the effective adoption of AI technologies in health care settings through multiple case studies, highlighting best practices and challenges	Diagnostic support, personalized medicine, patient monitoring, predictive analytics, and improving clinical workflows	Data privacy concerns, initial setup costs, need for high-quality training data, variability in model performance across different settings, resistance from health care professionals
Xie et al (2021) [35]		China	Chronic disease management	Qualitative study	To explore the integration of AI, blockchain, and wearable technology in managing chronic diseases, focusing on improving patient outcomes and optimizing health care delivery	Continuous monitoring, predictive analytics for disease progression, personalized treatment plans, secure data sharing, enhancing patient engagement	Data privacy concerns, initial setup costs, need for high-quality training data, variability in model performance across different populations, resistance from healthcare professionals
Yang et al (2022) [38]		United Kingdom	Gynecology	Cohort study	To develop and validate a ML-based risk stratification model for gestational diabetes management, aiming to improve patient outcomes through personalized risk assessment and intervention	Risk prediction, early detection, personalized treatment plans, improving clinical workflows, and enhancing patient engagement	Data privacy concerns, initial setup costs, need for high-quality training data, variability in model performance across different populations, resistance from health care professionals
Yoo et al (2022) [27]		Korea	Nonspecific	Clinical prediction	To develop and evaluate an interoperable and easily transferable clinical decision support system that can be effectively deployed across various health care settings, aiming to improve clinical workflows and patient outcomes	Diagnostic support, treatment planning, predictive analytics, improving clinical workflows, and enhancing patient safety	Data privacy concerns, initial setup costs, need for high-quality training data, variability in model performance across different settings, resistance from health care professionals
Wshah et al (2022) [24]		United States	Radiology	Diagnostic test study	To develop and validate a ML model for classifying intravascular volume status using point-of-care ultrasound, aiming to improve the accuracy of clinical assessments in critical care settings	Intravascular volume classification, diagnostic support, improving clinical decision-making, and enhancing patient outcomes	Data privacy concerns, initial setup costs, need for high-quality training data, variability in model performance across different populations, resistance from health care professionals
Tam et al (2021) [22]		United Kingdom	Radiology	Diagnostic test study	To evaluate how AI can assist radiologists as the first reader of chest x-rays, improving accuracy and efficiency in lung cancer diagnosis by triaging HCT^i^ cases before standard reporting	AI-based triage workflow; detection of lung nodules, masses, and hilar enlargement; reduction of missed cancers; standardization of radiologist performance; improved diagnostic consistency; enhanced performance on difficult or distracting findings	Increase in false positives, performance drop with distracting findings (eg, COPD^j^, pleural effusion), requires threshold tuning for HCT classification, algorithm not trained on local data
Seyam et al (2022) [46]		Switzerland	Radiology	Diagnostic test study	To evaluate the diagnostic performance and impact on clinical workflow of an AI-based tool for detecting ICH on emergent noncontrast head CT^l^ scans	Detection of various types of ICH^k^ (eg, intraparenchymal, subdural, subarachnoid, intraventricular), improved prioritization of critical findings, reduction in report turnaround times and ED^m^ length of stay	Lower detection rates for specific subtypes of ICH (eg, subdural and acute subarachnoid hemorrhage), false-positive findings (eg, postoperative defects, artifacts, tumors), need for clear standard operating procedures to ensure optimal functioning in patient care workflows
Raven et al (2022) [28]		The Netherlands	Emergency medicine	Clinical prediction	To evaluate whether ML combined with clinical judgment outperforms clinical judgment alone in predicting in-hospital mortality in both older and younger patients suspected of infection presenting to the ED	Risk stratification of ED patients with suspected infections, rapid initiation of appropriate treatment and disposition based on risk prediction, enhanced decision-making support for clinicians by integrating ML models into clinical workflows	Potential bias in training datasets. Need for validation in diverse populations. Integration challenges within existing clinical workflows. Possible overfitting if not properly validated
Hond et al (2021) [37]		The Netherlands	Emergency medicine	Clinical prediction	To develop and validate a ML model for predicting hospital admission in ED patients, aiming to improve patient flow and resource allocation	Prediction of hospital admission likelihood, optimization of patient triage and resource management, enhanced decision-making support for clinicians by providing real-time predictive analytics	Risk of overfitting the model to specific datasets, limited generalizability across different ED settings, potential bias due to missing data or unrepresentative sample, integration challenges with existing hospital information systems
Tuwatananurak et al (2019) [36]		United States	Surgery	Diagnostic test study	To evaluate whether ML models can improve the estimation of surgical case duration compared to traditional methods, aiming to optimize operating room scheduling and resource allocation	Accurate prediction of surgical case durations, optimization of operating room schedules, enhanced decision-making support for surgical planning and resource management, improved efficiency in hospital operations	Potential overfitting to specific datasets, limited generalizability across different surgical specialties or hospitals, Data quality issues such as missing or inaccurate data entries, integration challenges with existing hospital information systems
Bertsimas et al (2020) [41]		United States	Cardiology	Clinical prediction	To develop and validate a ML model to provide personalized treatment recommendations for patients with coronary artery disease, aiming to improve patient outcomes by optimizing treatment strategies	Personalized treatment recommendations based on individual patient characteristics, prediction of treatment effectiveness and adverse events, enhanced decision-making support for cardiologists, improved patient outcomes through optimized therapy selection	Potential overfitting to specific datasets, limited generalizability across different populations or health care systems, data quality issues such as missing or inaccurate data entries, ethical considerations regarding the use of ML in clinical decision-making, integration challenges with existing electronic health records systems

a AI: artificial intelligence.

b ICU: intensive care unit.

c ABPM: ambulatory blood pressure monitoring.

d MSK: musculoskeletal.

e PCOS: polycystic ovary syndrome.

f GDPR: General Data Protection Regulation.

g LVO: large vessel occlusion.

h ML: machine learning.

i HCT: high-confidence tumor.

j COPD: chronic obstructive pulmonary disease.

k ICH: intracranial hemorrhage.

l CT: computed tomography.

m ED: emergency department.

**Table 5.** Distribution of included studies by clinical domain, study design, and country of origin.

Category	Studies, n (%)
Clinical domain	
Nonspecific applications	10 (34.5)
Radiology	6 (20.7)
Emergency medicine	5 (17.2)
Gynecology	2 (6.9)
Cardiology	2 (6.9)
Surgery	1 (3.4)
Psychiatry	1 (3.4)
Nursing	1 (3.4)
Chronic disease	1 (3.4)
Study design	
Diagnostic test	8 (27.6)
Qualitative	6 (20.7)
Cohort	6 (20.7)
Clinical prediction	5 (17.2)
Cross-sectional	3 (10.3)
Economic evaluation	1 (3.4)
Country of origin	
United States	12 (41.4)
United Kingdom	3 (10.3)
The Netherlands	2 (6.9)
Korea	2 (6.9)
Italy	2 (6.9)
Germany	2 (6.9)
China	2 (6.9)
Uganda	1 (3.4)
Switzerland	1 (3.4)
India	1 (3.4)
France	1 (3.4)

In terms of study design, diagnostic test studies (8/29, 27.6%) [18,22-24,31,36,44,46] were the most common. Other study designs included cohort studies (6/29, 20.7%) [20,32,34,38,39,43], qualitative studies (6/29, 20.7%) [21,26,30,35,40,45], clinical prediction studies (5/29, 17.2%) [27,28,33,37,41], cross-sectional studies (3/29, 10.3%) [19,25,42], and economic evaluation studies (1/29, 3.4%) [29].

Geographically, the majority of studies originated from high-income countries (Table 6). The United States accounted for nearly half (12/29, 41.3%) [18,20,24,26,29,36,39-41,43-45], reflecting strong emphasis on EHR-linked AI, radiology, cardiology, and workflow optimization. European contributions included the United Kingdom (3/29, 10.3%) [22,34,38], Germany (2/29, 6.9%) [21,30], Italy (2/29, 6.9%) [19,42], France (1/29, 3.4%) [33], the Netherlands (2/29, 6.9%) [28,37], and Switzerland (1/29, 3.4%) [46], with common emphases on General Data Protection Regulation compliance, data sharing, and national cardiovascular initiatives. China contributed 2 studies (2/29, 6.9%) [25,35], reporting multicenter deployments (eg, DeepSeek across 90 tertiary hospitals) and AI integration with blockchain and wearable technologies. Korea contributed 2 studies (2/29, 6.9%) [27,32], focusing on nursing decision support and interoperable clinical decision support systems. Emerging economies were also represented: India (blockchain-enabled gynecology) [23] and Uganda (trauma triage in low-resource settings) [31]. Collectively, these geographic patterns demonstrate United States and European dominance but also highlight distinct implementation trajectories and challenges from Asia and other regions.

**Table 6.** National-level patterns of hospital AI^a^ Implementation.

Country/region	Studies, n	National/institutional strategy	Clinical focus	Reported benefits and barriers
China	2	Smart hospital initiatives, large-scale deployment	Multihospital imaging (DeepSeek); chronic disease management (AI+blockchain + wearables)	Improved decision support, high upfront costs, privacy/security concerns
United States	12	Data governance, interoperability, FDA^b^ oversight	Radiology, cardiology, emergency stroke, laboratory optimization, surgery	Strong EHR^c^-linked AI, adoption fragmented, dataset bias, explainability needs
United Kingdom	3	National programs, ethics, and privacy focus	Cardiovascular AI program, radiology triage, cohort, and risk models	Privacy governance emphasized, limited scale, implementation costs
The Netherlands	2	Hospital innovation pilots, workflow optimization	Emergency department prediction models (mortality, admission risk)	Improved triage and flow, generalizability limited, integration challenges
Korea	2	Interoperable CDSS^d^, generative AI in nursing	Nursing documentation, cross-setting CDSS	Workflow support, staff resistance, training data requirements
Italy	2	Efficiency and specialty focused	Energy management and psychiatry (ASD^e^ treatment)	Cost savings potential, privacy and acceptance challenges
Germany	2	Hospital AI adoption studies	Case-based AI adoption analysis	GDPR^f^ compliance and high-quality data needs
France, Switzerland	2	Specialty-specific pilots	ICU^g^ prediction (France); ICH^h^ detection (Switzerland)	Single-center focus and generalizability limits
India, Uganda	2	Low-/middle-income strategies	PCOS^i^ management with blockchain (India); trauma triage (Uganda)	Infrastructure limits and workforce adaptation

a AI: artificial intelligence.

b FDA: US Food and Drug Administration.

c EHR: electronic health record.

d CDSS: clinical decision support system.

e ASD: autism spectrum disorder.

f GDPR: General Data Protection Regulation.

g ICU: intensive care unit.

h ICH: intracranial hemorrhage.

i PCOS: polycystic ovary syndrome.

### Quality Assessment

The quality of the 29 studies was evaluated using the CASP standard. The studies comprised 6 study types: economic evaluations (n=1) [29], clinical prediction studies (n=5) [27,28,33,37,41], diagnostic test studies (n=8) [18,22-24,31,36,44,46], cohort studies (n=6) [20,32,34,38,39,43], qualitative studies (n=6) [21,26,30,35,40,45], and cross-sectional studies (n=3) [19,25,42]. Three studies (3/29, 10.3%) [20,23,34] scored below 40% of CASP items due to insufficient methodological descriptions and unclear recruitment or analysis procedures. Fifteen studies (15/29, 51.7%) [18,21,22,24,25,27,28,33,37,41-46] met between 50% and 70% of the criteria, whereas 11 studies (11/29, 37.9%) [19,26,29-32,35,36,38-40] exceeded 80%. In diagnostic and prediction studies, common limitations included incomplete reporting of recruitment processes, lack of external validation, and absence of blinding. Despite these weaknesses, most studies demonstrated clear research aims and appropriate methodological choices. Detailed CASP scores for each study are presented in Multimedia Appendix 3.

### RQ1 Findings

#### Overall Maturity Across the 5 Layers

To validate the proposed 5-layer architecture, all 29 studies were systematically mapped to the framework based on the maturity levels (Table 7). The application layer (mean 3.17, SD 0.85) and data layer (mean 3.00, SD 0.76) demonstrated the highest maturity, followed by the algorithm layer (mean 2.79, SD 0.77) and infrastructure (mean 2.79, SD 1.70) layers, the latter showing considerable variability across hospitals. Security and compliance layer (mean 1.69, SD 1.89) remained the least mature and most inconsistently addressed across studies. These findings suggest that research has higher maturity on data readiness, model development, and workflow integration, whereas infrastructure and governance considerations showed both lower maturity and greater variability, suggesting that technical capacity, institutional governance, and compliance mechanisms remain unevenly developed and inconsistently reported in primary studies. Detailed evidence for each mapping decision is provided in Multimedia Appendix 4.

**Table 7.** Five-layer evidence matrix.

Study	Infrastructure	Data	Algorithm	Application	Security and compliance
Ahsen et al [29]	0	2	2	2	0
Boussen et al [33]	0	3	3	2	0
Alam et al [18]	1	2	2	2	2
Areias et al [20]	4	3	3	4	0
Chen et al [25]	5	4	4	5	4
Farghaly and Deshpande [44]	2	2	3	2	0
Fairbairn et al [34]	5	4	4	4	4
Jaganathan and Natesan [23]	4	4	4	3	5
Muntasir et al [40]	4	4	3	4	3
Ju et al [32]	2	3	3	3	0
Klumpp et al [21]	1	2	2	2	3
Le et al [39]	4	3	3	4	0
Li et al [45]	4	3	3	4	0
Lin et al [43]	0	2	2	2	0
Novak et al [26]	0	2	0	3	2
Nsubuga et al [31]	0	2	3	2	0
Pariso et al [19]	4	3	3	4	0
Vignapiano et al [42]	3	3	2	3	0
Roppelt et al [30]	1	2	2	3	3
Xie et al [35]	4	4	3	3	5
Yang et al [38]	4	3	3	3	0
Yoo et al [27]	4	4	3	4	4
Wshah et al [24]	3	3	3	3	0
Tam et al [22]	4	3	3	4	4
Seyam et al [46]	4	3	3	4	4
Raven et al [28]	4	3	3	4	3
De Hond et al [37]	4	4	3	3	3
Tuwatananurak et al [36]	2	3	3	3	0
Bertsimas et al [41]	4	4	3	3	0
Mean (SD)	2.79 (1.70)	3.00 (0.76)	2.79 (0.77)	3.17 (0.85)	1.69 (1.89)

#### Evidence Stratified by Study Design

Given the methodological heterogeneity of the included studies, we conducted a stratified synthesis by study design (Table 8). The results of this analysis show that studies of clinical prediction, diagnostic test, and cohort studies achieved higher maturity in the data, algorithm, and application layers, particularly the clinical prediction studies, which showed the most consistent and advanced technical implementation. In contrast, qualitative and cross-sectional studies exhibited greater maturity variation, contributing more substantially to the infrastructure and security and compliance layers. The single economic evaluation demonstrated moderate maturity, limited mainly to the technical layers. This stratified synthesis highlights how methodological design shapes the visibility of different layers, with quantitative evaluation studies emphasizing technical robustness and data integration, whereas qualitative designs better capture infrastructural and governance maturity essential for sustainable AI platform development.

**Table 8.** Five-layer mapping stratified by study design.^a^

Design	Infrastructure, mean (SD)	Data, mean (SD)	Algorithm, mean (SD)	Application, mean (SD)	Security and compliance, mean (SD)
Clinical prediction	4.00 (0.00)	3.75 (0.50)	3.00 (0.00)	3.50 (0.58)	2.50 (1.73)
Cohort	3.17 (1.83)	3.00 (0.63)	3.00 (0.63)	3.33 (0.82)	0.67 (1.63)
Cross-sectional	4.00 (1.00)	3.33 (0.58)	3.00 (1.00)	4.00 (1.00)	1.33 (2.31)
Diagnostic test	2.50 (1.51)	2.75 (0.71)	3.00 (0.53)	2.88 (0.83)	1.88 (2.17)
Economic evaluations	0	2	2	2	0
Qualitative	2.00 (1.87)	2.80 (1.10)	2.00 (1.22)	3.00 (0.71)	3.20 (1.10)

a Values represent the mean (SD) of weighted maturity scores across studies of the same design type, calculated within each of the 5 layers of the proposed hospital artificial intelligence platform architecture: infrastructure, data, algorithm, application, and security and compliance. For the economic evaluations category, only 1 study was available; therefore, only the mean score is reported without SD.

#### Mapping the Evidence to the 5-Layer Framework

**Figure 3.**
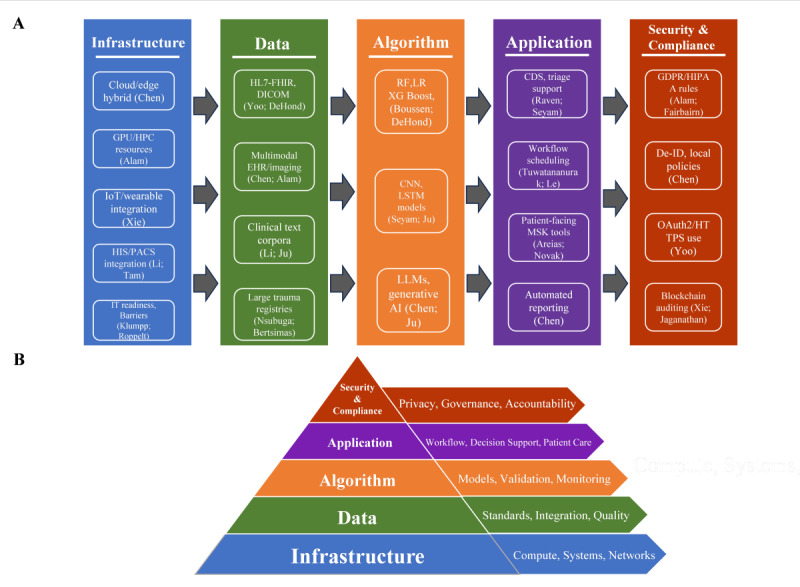
Overview and evidence mapping of the hospital AI platform architecture. (A) Extracted study-level findings were synthesized and organized within the 5-layer AI platform architecture. Each box summarizes commonly reported evidence elements, with exemplar studies cited in parentheses. (B) Simplified 5-layer pyramid showing broad categories (infrastructure: compute, systems, networks; data: standards, integration, quality; algorithm: models, validation, monitoring; application: workflow, decision support, patient care; security and compliance: privacy, governance, accountability). AI: artificial intelligences; CDS: clinical decision support; CNN: convolutional neural network; De-ID: deidentification; DICOM: Digital Imaging and Communications in Medicine; EHR: electronic health record; EMPI: Enterprise Master Patient Index; FHIR: Fast Healthcare Interoperability Resources; GDPR: General Data Protection Regulation; HIPAA: Health Insurance Portability and Accountability Act; HIS: health information system; HL7: Health Level Seven; IoT: Internet of Things; LLM: large language model; LR: logistic regression; LSTM: long short-term memory; MSK: musculoskeletal; PACS: picture archiving and communication system; RF: random forest.

We mapped the identified evidence to the 5-layer framework: (1) infrastructure layer, (2) data layer, (3) algorithm layer, (4) application layer, and (5) security and compliance layer. To achieve this, we reviewed the specific evidence in Multimedia Appendix 4 and selected high-frequency examples to map into the architecture. Figure 3A shows the detailed evidence mapping, where each layer is populated by distinct categories of evidence, and Figure 3B presents the simplified conceptual pyramid as an overview.

### RQ2 Findings

**Figure 4.**
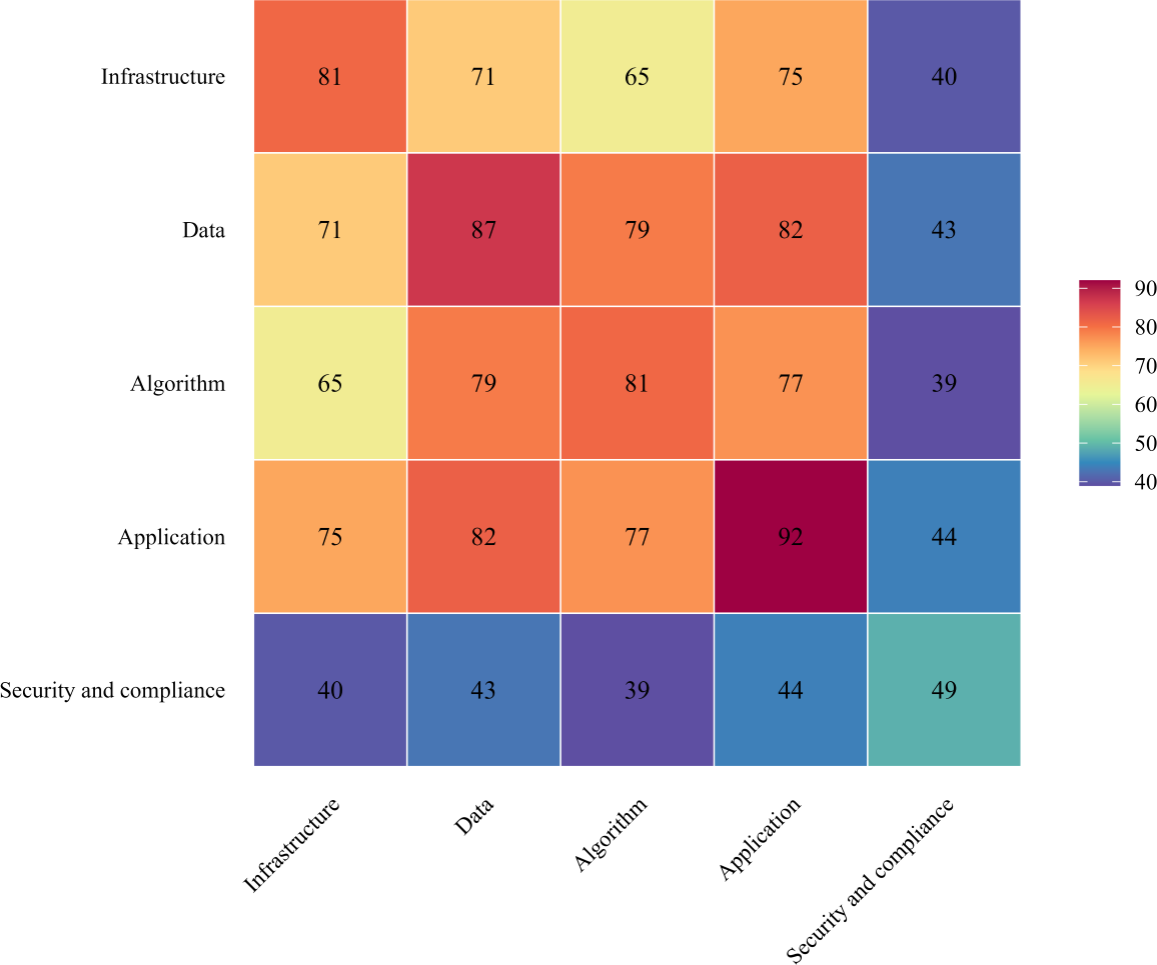
Weighted co-occurrence heatmap across the 5-layer architecture.

**Figure 5.**
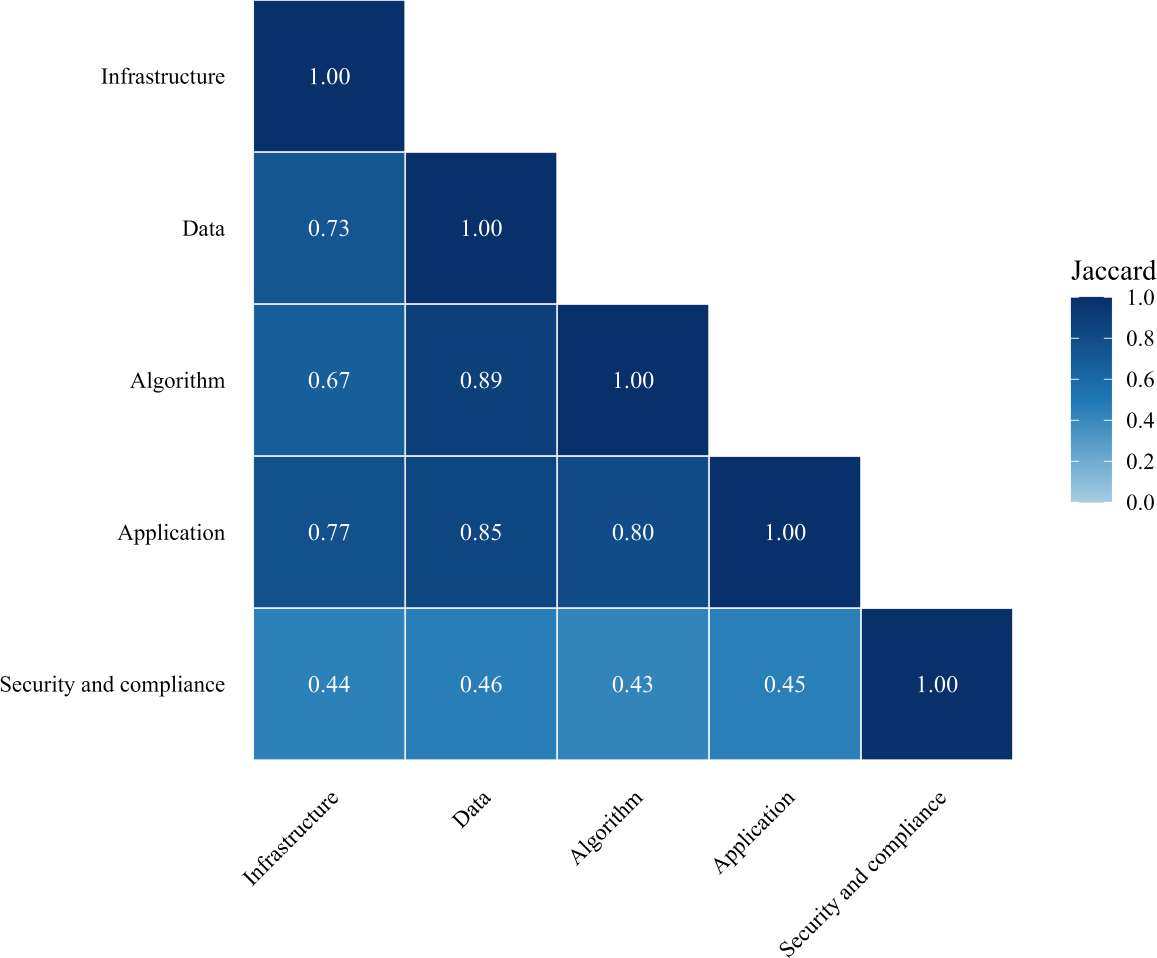
Weighted Jaccard similarity heatmap across the 5-layer architecture. Each cell represents the weighted Jaccard similarity between two layers across 29 included studies. Values were computed as the sum of the minimum maturity scores for each layer pair divided by the sum of their maximum scores. Higher values (darker shades) indicate stronger maturity alignment between layers. The data, algorithm, and application layers form a highly cohesive core (Jaccard=0.80‐0.89), whereas security and compliance exhibit weaker associations (Jaccard=0.43‐0.46), highlighting its peripheral integration in current hospital artificial intelligence implementations.

While RQ1 examined the maturity of individual layers, RQ2 explored the interrelationships among layers in hospital AI systems. The weighted co-occurrence heatmap (Figure 4) clearly shows that data, algorithm, and application exhibited the strongest interconnections in the center. The weighted Jaccard similarity heatmap (Figure 5) further confirmed this pattern, showing strong maturity overlap among the 3 layers (data-application=0.85, data-algorithm=0.89, and algorithm-application=0.80). This “core triad” indicates that data preparation, model development, and workflow integration are inseparable steps in most implementations.

Cells show weighted co-occurrence scores (sum of per-study maturity shared by each pair of layers, 0‐5 scale), so larger values indicate stronger cross-layer coupling. Diagonal cells give the cumulative maturity for each layer. The central triad, data, algorithm, and application, shows the strongest coupling (eg, data-application=82; data-algorithm=79; and algorithm-application=77), whereas security and compliance is consistently weaker with other layers. Warmer colors denote higher weighted coupling.

In contrast, security and compliance appeared peripheral, with lower scores when coupled with the other layers (Jaccard=0.43‐0.46). Infrastructure demonstrated moderate connectivity with application (Jaccard=0.77) and data (Jaccard=0.73), implying that infrastructural maturity often co-develops with technical capability but is not systematically aligned with governance or oversight mechanisms. These patterns point to the need for earlier integration of governance and compliance into platform design, ensuring they function as core rather than peripheral components.

### Implementation Examples

To assess if the proposed 5-layer hospital AI platform (infrastructure, data, algorithm, application, and security and compliance) can work in practice, we narratively synthesize 4 fielded deployments from our 29 included studies, in settings with explicit clinical integration and measurable end points.

#### Example 1

An AI application for noncontrast head computed tomography (CT) [46] was deployed in an emergency radiology setting to flag multiple intracranial hemorrhage subtypes and reprioritize critical cases in the radiology worklist. The deployed system, using PACS infrastructure (layer 1: infrastructure), which focused on curated CT datasets, engaged difficult cases, such as postoperative changes and artifacts (layer 2: data). A multisubtype detection algorithm (layer 3: algorithm) applied with balanced sensitivity and false positives allowing for better triage. This led to automated elevation of priority levels and reordering of queues clinically, which reduced turnaround times for report generation and emergency department length of stay (layer 4 application). To reduce the chances of false alarms being raised through human verification and not to miss out on any subtype, standard operating procedures were framed at this layer (layer 5: security and compliance).

#### Example 2

An AI-generated first-reader system was integrated into current workflows of chest radiographs to flag high-confidence tumor cases for earlier review [44]. The deployment was able to link with the digital radiography acquisition, PACS, Radiology Information System, reporting system for batch inference, and rule-based triggers (layer 1: infrastructure). Through the use of large-scale chest X-ray datasets in Digital Imaging and Communications in Medicine format with structured reads to calibrate thresholds and monitor downstream confirmations (layer 2: data). The software gave triage scores for nodules, masses, and hilar enlargement. These scores formed the high-confidence queue (layer 3: algorithm). At the application level, this facilitated reprioritization and consistency-oriented quality management across radiologists (layer 4: application), whereas false-positive audits and drift monitoring ensured compliance and performance stability over time (layer 5: security and compliance).

#### Example 3

A machine learning–enabled system at primary stroke centers aided in the early detection of large-vessel occlusion and accelerated transfer protocols [39]. Through CT and computed tomography angiography (CTA) acquisition, PACS, alerting on-call, and interhospital coordination platforms, the system was tightly coupled for smooth integration of infrastructure (layer 1: infrastructure). It combined imaging with time-stamped process data (arrival, transfer, reperfusion) to monitor pathway performance (layer 2: data). The algorithm automatically stratified large vessel occlusion cases, triggering alerts and activating predefined stroke pathways (layer 3: algorithm). At the application level, this enabled the rapid activation of “green channel,” transport prioritization, and standardization of decision points in acute stroke care (layer 4: application). As per the established transfer policies and accountability along the care chain, cross-site data sharing took place in a manner that governed the deployment according to the governance and compliance expectations (layer 5: security and compliance).

#### Example 4

A platform-scale AI deployment was rolled out across 90 tertiary hospitals to provide image analysis and clinical decision support at scale [25]. The infrastructure consisted of multisite compute and networking resources with containerized inference engines with full-stack EHR and PACS integration for seamless updates (layer 1: infrastructure). Data was curated across institutions, combining different types and standardization procedures as part of (layer 2: data) for use in other contexts. A portfolio of task-specific models was built as a platform asset with continuous iteration (layer 3: algorithm), providing triage, detection, recommendation, quality control, and more capabilities. The platform created common clinical entry points for decision support (layer 4: application) to streamline workflows across sites. Security and compliance were formalized through access control, audit trails, change management, and staff training, underscoring its readiness for large-scale operations (layer 5: security and compliance).

Across these examples, 3 cross-cutting themes emerged. First, workflow-native integration, such as worklist reprioritization, automated alerts, and transfer coordination, was the critical pathway for translating algorithmic outputs into measurable clinical benefits. Second, infrastructure and data robustness are critical to sustaining application-level improvements. Deployments without standard pipelines are fragile. Third, governance was put into action through standard operating procedures, threshold calibration, and audit mechanisms to control false positives, drift, and intersite variation. The 5-layer model is practically viable and also highlights where further investment is needed, including in infrastructure resilience, data governance, and clinician adoption.

## Discussion

### Principal Findings

A 5-layer hospital AI platform model (infrastructure, data, algorithm, application, and security and compliance) was developed by synthesizing 4 reference frameworks. From 283 records screened, 29 studies (29/283, 10.2%) were included with high interrater reliability (κ=0.98). Most studies were diagnostic test (8/29, 27.6%) or qualitative (6/29, 20.7%), and almost half were conducted in the United States (12/29, 41.4%). Quality varied: only 37.9% (11/29) of studies achieved more than 80% of CASP items, whereas 10.3% (3/29) scored below 40%.

Evidence mapping showed the application (mean 3.17, SD 0.85), data (mean 3.00, SD 0.76), and algorithm (mean 2.79, SD 0.77) layers have the highest and most balanced maturity, forming a tightly integrated core triad. Weighted co-occurrence analysis demonstrated the strongest interconnections among these 3 layers (data-application=82, data-algorithm=79, and algorithm-application=77), and weighted Jaccard similarity indices confirmed substantial maturity overlap (data-algorithm=0.89, data-application=0.85, and algorithm-application=0.80). The infrastructure layer (mean 2.79, SD 1.70) displayed moderate maturity but high variability. The security and compliance layer (mean 1.69, SD 1.89) remained the least mature and weakly connected to the others (Jaccard=0.43‐0.46). Stratification by study design showed that technical studies (diagnostic, prediction, and cohort) achieved higher maturity within the technical core, whereas qualitative and cross-sectional studies more often addressed infrastructure and governance that remain underdeveloped in most technical evaluations.

### Comparison With Prior Work

There was a maturity imbalance across the layers, which were dominated by technical layers. There were similar trends observed in earlier reviews, which showed that algorithm performance and workflow integration achieved higher maturity, whereas infrastructure, ethics, and compliance were less developed [47,48]. Several factors may explain this. The focus of studies and publication bias has favored predictive accuracy and model validation [47,49]. Most studies were led by clinical and technical teams. The governance and IT planning teams were featured less due to disciplinary divides [50]. Many studies described pilot projects, where compliance and infrastructure emerged later in scaling.

Domain-specific differences were noted. In emergency imaging [39,46], infrastructure and application layers were highly mature due to the urgency of real time, whereas compliance was limited to operating procedures. In routine imaging [22,44], data-algorithm dominated, with application relying on threshold tuning. In chronic disease management [38,41], data governance and compliance were critical for privacy-preserving integration, and application focused on longitudinal risk stratification. In deployments across multiple hospitals [25], infrastructure and data pipelines were key bottlenecks, and compliance was institutionalized through audit trails and training. Negative outcomes were also reported: chest radiograph triage increased false positives in patients with comorbidities [22], ICH detection struggled with specific subtypes [46], and multihospital systems faced high costs and low adoption [25]. These findings confirm that success depends on context, workflow fit, and governance.

### Implications for Practice and Policy

Administrators can use the 5-layer model for phased investments, particularly in the areas of infrastructure and compliance, to eradicate blockages at a later stage [51,52]. Policymakers can promote compliance-by-design standards, requiring privacy, accountability, and explainability from the outset [53,54]. Vendors can align products with hospital priorities by embedding interoperability and monitoring tools [52]. Funding organizations and health systems should support the underdeveloped layers, especially governance and infrastructure through training and cross-institutional collaboration [51].

### Challenges and Limitations of AI Deployment in Hospitals

Technical and infrastructural barriers were widely reported. Data were often fragmented across EHR, LIS, and PACS, and many systems lacked application programming interface support or sufficient computing capacity [55,56]. These weaknesses limited scalability, whereas bandwidth bottlenecks reduced real-time performance and inadequate monitoring allowed model drift [22,25,46]. Solutions that have been proposed include HL7 FHIR–based data lakes [57], hybrid cloud-edge architectures [58], and continuous monitoring with automated retraining pipelines [59,60].

Organizational and adoption barriers also affected implementation. Clinician skepticism, workflow misalignment [22,46], and lack of structured feedback were common problems [30,35,61,62]. Better outcomes were described when interdisciplinary teams were formed, clinical champions were engaged early, and projects targeted low-risk and high-value use cases [63,64].

Regulatory and compliance gaps were identified as the weakest layer. Privacy safeguards, governance frameworks, and liability protocols were rarely embedded into early projects [21]. Compliance-by-design approaches have been recommended, supported by federated learning [65], explainability mechanisms, and blockchain-based audit trails [66], together with early engagement of regulators [67].

Economic and resource barriers were another critical concern. High upfront and maintenance costs, uncertain return on investment, and misalignment with hospital budget cycles were repeatedly described [25,68]. Strategies such as phased investments, AI-as-a-service models, and shared consortia were suggested to reduce costs and support sustainable deployment [69].

### Limitations of This Review and Future Research

This review has several limitations. A limitation is that only peer-reviewed English-language publications were included, which may have excluded valuable implementation reports published in other languages or as gray literature. This restriction was applied to maintain consistency and reliability in the 0 to 5 ordinal maturity scoring, as translation variability could compromise coding accuracy and interrater agreement. In addition, most gray literature lacks formal peer review or standardized reporting, which may introduce methodological inconsistency and compromise the overall reliability of evidence synthesis. Therefore, it was intentionally excluded to preserve data quality and comparability. Another limitation is that studies were mapped to the 5-layer framework using an ordinal maturity scoring, which may not fully reflect the complexity of AI applications. A further limitation is that the framework was validated only through literature synthesis and has not been prospectively tested in real hospital environments, such as in resource-limited settings or smaller hospitals.

Future research should extend evidence retrieval to include non-English and gray literature through calibrated multilingual screening and curated institutional sources. Further refinement of maturity metrics and cross-layer evaluation methods is warranted to better represent the dynamic evolution of hospital AI systems. Furthermore, prospective multicenter studies are also required to validate the framework in practice and to test its scalability across diverse hospital settings.

### Conclusions

The integration of hospital information systems with AI is essential for the digital transformation of health care. Evidence from 29 empirical studies was synthesized with established frameworks to validate a 5-layer architecture. This model provides both theoretical and practical guidance for platform-level AI in hospitals. The framework can be applied by researchers, practitioners, and policymakers to support the development of scalable, secure, and clinically integrated AI platforms.

## Supplementary material

10.2196/79788 Multimedia Appendix 1 Search strategy and strings for all databases.

10.2196/79788 Multimedia Appendix 2 The 2×2 contingency table used for interrater reliability calculation.

10.2196/79788 Multimedia Appendix 3 Detailed results of the Critical Appraisal Skills Programme quality assessment for included studies.

10.2196/79788 Multimedia Appendix 4 Detailed evidence mapping of all included studies to the 5-layer framework.

10.2196/79788 Checklist 1 PRISMA 2020 checklist.

## Abbreviations

AI: artificial intelligence
CASP: Critical Appraisal Skills Programme
CT: computed tomography
EHR: electronic health record
FHIR: Fast Healthcare Interoperability Resources
HIS: Hospital information system
ICH: intracranial hemorrhage
IoT: Internet of Things
LIS: laboratory information system
ML: machine learning
PACS: picture archiving and communication system
PRISMA: Preferred Reporting Items for Systematic Reviews and Meta-Analyses
