# Supplementary material for: Artificial Intelligence Platform Architecture for Hospital Systems: Systematic Review
Source: J Med Internet Res. 2025 Dec 17;27:e79788. doi: 10.2196/79788 (PMC12710730; doi:10.2196/79788)
Supplement: Multimedia Appendix 1 [file jmir-v27-e79788-s001.docx]

Table. Search strategy and strings for Web of Science, EMBASE, PubMed and Scopus.

| **ID** | **Search strings** |
| --- | --- |
| **#1** | Hospital Management |
| **#2** | Healthcare Management |
| **#3** | Hospital Operations |
| **#4** | Healthcare Administration |
| **#5** | Medical Administration |
| **#6** | Hospital Information Systems |
| **#7** | #1 OR #2 OR #3 OR #4 OR #5 OR #6 |
| **#8** | AI Deployment |
| **#9** | AI Implementation |
| **#10** | AI Integration |
| **#11** | #8 OR #9 OR #10 |
| **#12** | #7 AND #11 |
| **#13** | Artificial Intelligence |
| **#14** | AI Applications |
| **#15** | AI |
| **#16** | Large Language Model |
| **#17** | Transformer applications |
| **#18** | #13 OR #14 OR #15 OR #16 OR #17 |
| **#19** | Hospital Operations Optimization |
| **#20** | Clinical Workflow Improvement |
| **#21** | Resource Allocation |
| **#22** | Patient Flow Management |
| **#23** | #2 OR #19 OR #20 OR #21 OR #22 |
| **#24** | #18 AND #23 |
| **#25** | #12 AND #24 |
