# Supplementary material for: Artificial Intelligence Platform Architecture for Hospital Systems: Systematic Review
Source: J Med Internet Res. 2025 Dec 17;27:e79788. doi: 10.2196/79788 (PMC12710730; doi:10.2196/79788)
Supplement: Multimedia Appendix 2 [file jmir-v27-e79788-s002.docx]

Table. Overall reviewer agreement in study selection

|  | Reviewer 2: Include | Reviewer 2: Exclude | Row total |
| --- | --- | --- | --- |
| Reviewer 1: Include | 93 | 1 | 94 |
| Reviewer 1: Exclude | 2 | 159 | 161 |
| Column total | 95 | 160 | 255 |

Note. The 2×2 table summarizes the pre-consensus independent screening results at the title/abstract stage (n = 255). Both reviewers initially agreed to include 93 records, while 3 records were considered borderline cases that led to differing initial judgments. These discrepancies mainly involved studies that partially met the inclusion criteria but required clarification on the extent of hospital involvement or data origin. After discussion, both reviewers agreed that these studies were relevant to hospital-level AI implementation and therefore qualified for inclusion. As a result, 96 records were ultimately advanced to full-text review, reflecting consensus resolution of the three borderline cases and confirming the high inter-rater reliability (Cohen’s κ = 0.98).
