# Supplementary material for: Artificial Intelligence Platform Architecture for Hospital Systems: Systematic Review
Source: J Med Internet Res. 2025 Dec 17;27:e79788. doi: 10.2196/79788 (PMC12710730; doi:10.2196/79788)
Supplement: Multimedia Appendix 4 [file jmir-v27-e79788-s004.docx]

Table. Comprehensive evidence mapping of all included studies to the five-layer framework

| **Author** |  |  | **Evidence** |  |  |
| --- | --- | --- | --- | --- | --- |
|  | **Infrastructure Layer** | **Data Layer** | **Algorithm Layer** | **Application Layer** | **Security and Compliance Layer** |
| Mehmet Eren Ahsen | Not discussed | NHS Breast Screening Programme data used for modeling: includes recall rates, cancer incidence, etc. | AI performance metrics (sensitivity/specificity) explicitly modeled; used to simulate decision-making flow | Multiple AI-human task-sharing workflows modeled for operational deployment in screening programs | Not discussed |
| S.Boussen | Not discussed | Real-time ECG signal data from ICU; heart rate variability and complexity features extracted for model input | Used multiple machine learning models: XGBoost, Logistic Regression, K-Nearest Neighbors, MLP; HRV features fed into classifiers | Application scenario is ICU mortality prediction; model performance compared to SAPS II and assessed via AUC scores | Not discussed |
| Sreyoshi F. Alam | Refers to the necessity of integrated Electronic Health Record (EHR) platforms and high-performance computing resources (e.g., GPU acceleration) for personalized medicine, but lacks specific technical deployment details. | Emphasizes the importance of data-driven approaches, including multi-omics data (genomics, proteomics), electronic health records, remote monitoring data, and sensor data. | Reviews and analyzes multiple AI algorithms used in disease risk prediction, treatment recommendation, and drug response modeling, such as deep learning, machine learning, and reinforcement learning. | Provides specific application scenarios such as diabetes management, precision oncology, chronic disease prediction, and individualized drug therapy planning. | Discusses issues of data privacy, GDPR compliance, algorithmic bias, and emphasizes the need for privacy-preserving modeling and ethical frameworks. |
| Anabela C. Areias | The platform is built to support remote care via digital devices, enabling AI-driven assessment and monitoring. It includes integration with patient interfaces and cloud-based delivery systems. | Uses patient-reported data, exercise adherence metrics, pain/function scores, and continuous progress tracking collected through the platform. | AI components guide initial assessment, triage, and adaptive exercise planning using patient data and engagement trends. | Applied for real-world MSK management, including diagnosis support, patient engagement, education, and feedback loops. | Not discussed |
| Jishizhan Chen | Describes deployment across 90 hospitals with cloud–edge hybrid architecture, secure API interfaces, and integration into HIS systems. | Utilizes multimodal patient data: EHR, lab tests, images, and conversational inputs captured from clinicians and patients. | Focuses on LLM-based NLP models (DeepSeek) fine-tuned on Chinese clinical corpora, supporting clinical reasoning, summarization, and instruction-following. | Real-world applications include automatic documentation, diagnostic support, medical Q&A, clinical decision support, and workflow automation. | Describes security standards for model deployment, de-identification measures, and adherence to Chinese data protection regulations. |
| Omar Farghaly | Describes use of CT imaging devices and integration of medical image databases into computing systems for model training and testing. | Uses public CT datasets (e.g., SARS-CoV-2 vs pneumonia) and extracts GLCM texture features from images. | Applies ML algorithms (Random Forest, SVM, k-NN) to classify texture-based features. No deep learning used. | Targeted application is assisting clinicians in distinguishing COVID-19 from viral pneumonia in CT images to aid faster diagnosis. | Not discussed |
| Timothy A. Fairbairn | Describes deployment of AI-enabled systems across multiple hospitals, cloud-based analytics platforms, and EMR integration at a national scale. | Utilized real-world clinical datasets, including structured EMRs, diagnostic imaging, and administrative data from multiple healthcare institutions. | Applied validated deep learning and machine learning models to predict 1-year cardiovascular mortality risk and system performance metrics. | Used AI to prioritize care pathways, enable targeted resource allocation, and support physician decision-making in cardiovascular risk management. | Outlined involvement of legal and policy stakeholders, governance frameworks, and bias evaluation, especially concerning indigenous and marginalized populations. |
| Gowthami Jaganathan | System architecture includes cloud-based blockchain deployment and federated machine learning setup across multiple institutions. | Utilizes clinical data including hormonal profiles, ultrasound reports, and patient history; addresses distributed and secure data handling via blockchain. Supported: Yes | Applies explainable ML models (e.g., SHAP, LIME) and federated learning to maintain privacy; includes neural network models for PCOS prediction. | Designed for early PCOS detection, multi-center collaboration, and feedback to clinicians with interpretable AI output for informed diagnosis. | Employs blockchain for immutable records, access control, and audit trails; specifically addresses patient data ownership and GDPR-aligned governance. |
| Jasmine Muntasir | Evidence: Describes integration with existing Laboratory Information Systems (LIS) and deployment across multiple hospital sites. | Real-time acquisition of lab test orders, patient identifiers, time stamps, and operational metadata from hospital systems. | Uses machine learning (ML) models for predicting optimal technician scheduling, resource allocation, and turnaround time estimation. | Applied to daily lab operations such as sample batching, triaging of urgent tests, and improving lab throughput. | Discusses secure data integration and anonymization, though not extensively; compliance with institutional ethics and data governance was acknowledged. |
| Hongshin Ju | Deploys a generative AI model (GPT-based) on a platform that integrates with virtual EMRs; however, lacks detail on underlying cloud or compute infrastructure. | Inputs include structured V-EMR content (symptoms, vitals, diagnoses, history); used to simulate real clinical records for model input. | Uses transformer-based LLMs (GPT-like) trained on nursing diagnosis and documentation standards to generate recommended outputs. | Generates nursing diagnoses, documentation drafts, and guides user feedback loops; demonstrates improved efficiency and standardization. | Not discussed |
| Matthias Klumpp | Discussed the need for AI-ready IT infrastructure, including interoperable systems, data centers, and readiness for cloud deployment across EU hospitals. | Emphasized data sharing across European hospitals, the use of federated learning, and interoperability of EHR systems for cross-institutional datasets. | Detailed application of machine learning and deep learning for diagnostic and triage systems in ICU, radiology, and early warning systems. | Listed concrete clinical implementations including automated triage, ICU monitoring, sepsis prediction, and radiology workflow optimization. | Extensively addressed GDPR, data pseudonymization, patient consent, and the ethical framework for explainable and trustworthy AI in EU healthcare. |
| Ngoc Mai Le | Used cloud-hosted AI platform integrated into primary stroke centers and spoke hospitals for LVO detection and transfer coordination. | Utilized CT angiogram imaging data and transfer times data from multiple hospital systems for model validation. | Applied deep learning algorithms to detect large vessel occlusion (LVO) from CT angiograms with high sensitivity. | Enabled earlier LVO detection and faster transfers, improving workflow in stroke networks and reducing time to treatment. | Not discussed |
| Zuotian Li, MS | Cloud-based AI infrastructure integrated into hospital systems, with real-time visualization dashboard (TrajVis) accessible through web browsers. | Extracted AI-predicted patient trajectory data and longitudinal EHR data; used these to train and validate clinical trajectory prediction models. | Implemented ensemble machine learning models to predict outcomes; used explainable AI to identify trajectory features. | Visual decision support for clinicians using a web-based system (TrajVis) to explore, interpret, and apply patient trajectory predictions. | Not discussed |
| Yannan Lin | Not discussed | The simulation utilized real-world patient data and mammography recall data to evaluate outcomes under different scheduling scenarios. | Risk-stratification algorithms were used to group patients and simulate the impact on recall rates and appointment wait times. | The simulation demonstrated practical application for improving recall workflows and resource allocation in clinical screening settings. | Not discussed |
| Laurie L. Novak | Not discussed | Project HealthDesign involved creating personal health records (PHRs) integrating patient-generated data such as symptoms, sleep, and medication use. | Not discussed | Strongly supported. Multiple prototypes were developed for patients and clinicians, including visualization tools and mobile interfaces. | There was mention of regulatory barriers (e.g., HIPAA limitations) when storing and sharing PHR data, highlighting compliance issue |
| Mike Nsubuga | Not discussed | Trauma registry data from Mulago National Referral Hospital in Uganda was used, including patient demographics and physiological variables. | Multiple ML models were trained and compared, including DNN, Random Forest, and XGBoost, to predict patient outcomes. | The models were designed to support trauma triage decisions in low-resource settings, identifying severely injured patients needing urgent care. | Not discussed |
| Paolo Pariso | The study discusses the deployment of IoT sensors, smart meters, and building management systems (BMS) to support AI-powered energy management. | Time-series data on energy usage, temperature, equipment load, and operational schedules were collected for AI model training. | AI methods including reinforcement learning, predictive analytics, and clustering were applied to forecast demand and control energy usage. | The models were integrated into hospital energy systems to manage HVAC and lighting in real-time for efficiency gains. | Not discussed |
| Annarita Vignapiano | The study developed a web-based platform integrating GIS tools, spatial services, and remote access infrastructure for ASD service mapping. | The system integrated clinical data, patient demographic and diagnostic data, as well as geographic location and provider information. | Machine learning techniques (e.g., KNN classification) were used to recommend and rank appropriate services based on proximity and patient needs. | The platform offered personalized recommendations, interactive maps, and real-time service matching for patients and families. | Not discussed |
| Julia S. Roppelt | Several case sites identified barriers in computing infrastructure, including legacy systems, limited computational power, and integration limitations. | Data quality, structuring, annotation, and interoperability were recurring concerns across organizations. | AI tools used include image recognition algorithms, NLP systems, and risk prediction models. | AI was used in clinical workflows for diagnostic support, triage, administrative automation, and communication. | The study discusses GDPR, model bias, ethical considerations, and clinical accountability in AI deployment. |
| Yi XIE | The platform uses IoT infrastructure integrated with wearable devices and edge computing to capture and transmit real-time health data. | Physiological signals such as heart rate, blood pressure, and motion are collected from wearables and stored securely on a blockchain. | AI models are applied to assess risk, detect abnormal patterns, and provide health insights based on wearable data. | Supports chronic disease management through alerts, medication reminders, and continuous health monitoring functionalities. | Blockchain ensures data integrity, tamper-resistance, and controlled access to sensitive health information. |
| Jenny Yang | EHR infrastructure was used to collect and integrate clinical data, with ML models embedded for inference. | Clinical variables including demographics, laboratory tests, and obstetric history were extracted for model development. | Random Forest, XGBoost, and Logistic Regression were used for GDM risk stratification. Model performance was compared and validated. | Models provided risk stratification to assist clinicians in decision-making for gestational diabetes management. | Not discussed |
| Junsang Yoo | The framework supports both cloud-based and on-premise deployments, allowing flexible integration across hospital environments. | The system is built on HL7 FHIR standards, ensuring standardized data representation and interoperability. | Supports integration of existing AI models (e.g., deep neural networks) through REST APIs to perform clinical inferences. | Clinical functions such as medication recommendations, alerts, and decision support are integrated into physician workflows. | Implements OAuth2 authorization and HTTPS communication for secure access and data exchange. |
| Safwan Wshah | Bedside ultrasound devices were used to capture IVC images, which were then uploaded to a central processing system. | Collected B-mode ultrasound images labeled with clinician-identified volume status for training and evaluation. | Convolutional Neural Networks (CNNs) were developed and trained to classify volume status based on ultrasound images. | The system is intended to assist fluid management decisions at the bedside based on AI-generated classifications. | Not discussed |
| M.D.B.S. Tam | AI model was integrated with the hospital PACS and IT systems, allowing seamless image retrieval and inference. | Training data consisted of historical chest radiographs labeled with benign and malignant lung nodules. | Deep convolutional neural networks were used to detect nodules and calculate malignancy scores. | AI-assisted radiologists in detecting malignancies, improved report speed and reduced diagnostic errors. | Study was approved by the ethics committee and conformed to data privacy regulations. |
| Muhannad Seyam | AI model was integrated into the hospital's PACS and functioned in real-time alongside emergency CT workflow. | Thousands of annotated non contrast head CT images were used to train and evaluate the AI model across multiple ICH types. | A deep learning model detected intracranial hemorrhage and generated visual overlays to highlight affected areas. | AI provided pre-read alerts, triaged positive cases for priority review, and supported diagnostic decision-making. | The study followed HIPAA standards and received IRB approval for data usage and integration. |
| Wouter Raven | ML models were integrated with the clinical research platform and emergency department workflow in real time. | EHR data from 12,844 emergency department patients were used, including demographics, vitals, labs, and clinical observations. | Multiple ML models including CatBoost, XGBoost, Random Forest, and Logistic Regression were trained and evaluated. | ML-assisted clinical judgment outperformed both ML alone and clinicians alone in predicting hospital admissions. | IRB approval was obtained and patient data were anonymized to comply with privacy regulations. |
| De Hond | Hospital information systems were used to extract standardized input variables. The model was externally validated across five hospitals. | EHR data from 66,000+ ED patients from five hospitals, including demographics, vital signs, chief complaints, etc. | Models compared include Logistic Regression, Random Forest, XGBoost, and LightGBM. LightGBM was selected as best. | Model used to predict admission risk at patient arrival, aiding decisions. SHAP used for explainability. | Data anonymized and IRB approval obtained for secondary use of healthcare data. |
| Justin P. Tuwatananurak | Historical surgical data were extracted from hospital IT systems and proposed for integration into OR scheduling systems. | 9,654 surgical cases used with 21 variables including procedure type, surgeon, anesthesia type. Data sourced from hospital HIS. | Multiple models trained including SVR, MLP, Random Forest, and XGBoost. Accuracy assessed at ±10, ±20, ±30 min intervals. | Models aimed to assist surgical scheduling by improving case duration prediction and reducing delays. | Not discussed |
| Dimitris Bertsimas | Built on institutional EHR data platform; the modeling framework supports deployment at point of care. | Over 50,000 CAD patients' structured clinical records, including demographics, treatment, and outcomes from EHR and registry data. | Applied Random Forests, Gradient Boosting, and CART to simulate outcomes across treatment strategies (CABG, PCI, Medical Therapy). | Used ML output to assign optimal personalized treatment strategy for each patient to improve long-term survival. | Not discussed |
